# Effect of Flaxseed Supplementation in Diet of Dairy Cow on the Volatile Organic Compounds of Raw Milk by HS-GC–IMS

**DOI:** 10.3389/fnut.2022.831178

**Published:** 2022-02-14

**Authors:** Guoxin Huang, Ning Li, Kaizhen Liu, Jiyong Yang, Shengguo Zhao, Nan Zheng, Jinhui Zhou, Yangdong Zhang, Jiaqi Wang

**Affiliations:** ^1^Key Laboratory of Quality and Safety Control for Milk and Dairy Products of Ministry of Agriculture and Rural Affairs, Institute of Animal Sciences, Chinese Academy of Agricultural Sciences, Beijing, China; ^2^State Key Laboratory of Animal Nutrition, Institute of Animal Sciences, Chinese Academy of Agricultural Sciences, Beijing, China; ^3^College of Animal Sciences and Technology, Northeast Agricultural University, Harbin, China; ^4^Institute of Food Science and Technology, Chinese Academy of Agricultural Sciences, Beijing, China

**Keywords:** flaxseed, milk, volatile organic compounds, HS-GC-IMS, n-3 PUFA

## Abstract

Flaxseed supplementation in diet of dairy cow can effectively enhance the production of ω-3 polyunsaturated fatty acids (n-3 PUFA) in raw milk, which further give rise to the changes of volatile organic compounds (VOCs). In this study, we used headspace-gas chromatography-ion mobility spectrometry (HS-GC-IMS) to investigate the VOCs in milk from cows fed three different diets (CK: supplemented with 0 g/d flaxseed; WF: 1,500 g/d whole flaxseed and GF: 1,500 g/d ground flaxseed). A total of 40 VOCs including three acids, six esters, 11 aldehydes, seven alcohols, 13 ketones were identified in all the raw milk samples. Compared with GF supplementation, suppling with WF could influence more compounds in raw milk (GF: five compounds; WF: 22 compounds). Supplementation with WF could increase the concentration of nonanal, heptanal, hexanal, which could cause the occurrence of off-flavors, and reduce the concentration of hexanoic acid (monomer; M), 2-hexanol, ethanol (M), 2-heptanone (dimer; D), 2-pentanone (M), 2-pentanone (D), acetoin (M) in raw milk. GF supplementation in diet could reduce the 2-pentanone (M), 2-pentanone (D). In addition, principal component analysis (PCA) based on the signal intensity of identified VOCs indicated that it is possible to distinguish between the CK and WF milk. However, GF milk could not be distinguished from CK milk. The results demonstrate that compared with GF milk, WF supplementation in diet of dairy cows could increase fishy (heptanal) cardboard-like (pentanal) flavor in milk and decrease sweet (hexanoic acid, 2-heptanone), fruity (ethyl butanoate, ethyl hexanoate, 2-heptanone) flavor which may lead the milk less acceptable. In conclusion, compared with WF, GF supplementation in diet of dairy cow showed higher increase in n-3 PUFA in raw milk, and less influence in VOCs of raw milk and this study might provide theoretical supports for the production of milk rich in n-3 PUFA.

## Introduction

ω-3 polyunsaturated fatty acids (n-3 PUFA) are essential nutrients for humans, and play a positive role in both the prevention and treatment of diseases, such as cardiovascular disease, cancer, diabetes, and kidney disease (1), and nervous system development (2). Therefore, the development of milk rich in n-3 PUFAs to improve the human health has attracted increasing attention in animal production. As flaxseed is rich in n-3 PUFA (3), it is often used as a source of n-3 PUFA in the diets of dairy cows to increase the content of n-3 PUFA in milk (4). Hence, whole flaxseed (WF) (5) and ground flaxseed (GF) (6) were introduced to the diets of dairy cows to enrich the level of n-3 PUFA in milk.

Previous reports stated that many factors such as feed (7) and breed (8) can cause variations in the volatile organic compound (VOC) levels in milk. Most studies that investigate feed focused on the forage types (7, 9). For However, no studies reported the effects of flaxseed supplementation on milk flavor. A study on eggs found that supplementation with flaxseed can cause an off-flavor in eggs (10, 11). And the α-linolenic acid (ALA, a kind of n-3 PUFA) in diet is mainly factor that leading to the off-flavor. Previous reported that different forms of flaxseed release ALA by different mechanisms in the vivo (12). Thus, different form flaxseed supplementation may also lead to the different sensory qualities and consumer acceptance.

In recent years, the most commonly used methods for the separation and identification of volatile components in milk include chromatography-mass spectrometry (GC-MS) (13), pulsed flame photometric detection (GC-PFPDs) (14), and flame ionization detection (GC-FIDs) (15) coupled with olfactometry. Ion mobility spectrometry (IMS) is a gas-phase separation and detection technique to analyze ionic substances based on their difference of migration rate of gas phase ions in electric field (16). IMS combined with GC can improve separation capability for resolving complex matrices in ionization and separation regions, and increase the amount of information collected from analytes (17). Owing to its advantages including high sensitivity (ppbv level), ultra-high analytical speed (3–10 s per sample), simplicity and ease of operation at atmospheric pressure (without excessive manual intervention), better separation in two dimensions and color contours image to display the differences among various samples, Headspace gas chromatography-ion mobility spectrometry (HS-GC-IMS) has been widely used for food flavor detection (18), such as for ham (19), garlic (20), honey (21) and rice (22). For milk, many articles used by IMS focused on dairy product such as milk powder (23), milk kefir (24), cheeses (25). However few studies have reported the use of IMS to analyze flavor compounds in raw milk.

Thus, the aim of this study was to investigate the effects of flaxseed supplementation in diet of cow on VOCs in raw milk. Three diets were tested in this experiment: 0 g/d flaxseed (CK), 1,500 g/d WF, and 1,500 g/d GF, and cows were fed a total mixed ration. The VOCs in raw milk from the three groups were investigated using headspace-gas chromatography-ion mobility spectrometry (HS-GC-IMS). The identification of VOCs in this study provided a theoretical basis for the development of n-3 PUFA-rich milk to exert a variety of beneficial human health effects.

## Materials and Methods

### Raw Milk

Raw milk samples were obtained from Tianjin Fuyou Agricultural Technology Co., Ltd (Tianjin, P.R. China) from 30 Holstein dairy cows (mean ± standard deviation; 90 ± 28 days in milk and 628 ± 103 kg body weight), from July to September. The dairy cows were randomly divided into three group, and fed three different diets. The composition of diets was were reported in our previous study (12). Briefly, the cows fed no flaxseed (CK) or fed a 1,500 g/d whole flaxseed supplementation diet (WF) or 1,500 g/d ground flaxseed supplementation diet (GF). The diets in this experiment were formulated and evaluated using the Feeding Standards of Dairy Cattle in P.R. China (Ministry of Agriculture of P.R. China (MOA), Feeding standard of dairy cattle, NY/T 34-2004; MOA: Beijing, P.R. China, 2004.). This experiment lasted 5 weeks. The cows were housed in a well-ventilated barn and fed individually, and feed three times per day (at 06:00, 12:00, and 21:30 h). The milk samples were collected at the day end of experiment, and according to the morning, afternoon, and night (milking volume ratio of 4:3:3). The raw milk stored at 4°C for HS-GC–IMS analysis. In this experiment, compared to WF milk, GF supplementation in diet of dairy cow showed higher increase in n-3 PUFA in raw milk.

### HS-GC–IMS Analysis

Raw milk samples were analyzed using an IMS instrument (FlavourSpec®, Gesellschaft für Analytische Sensorsysteme mbH, Dortmund, Germany) equipped with an autosampler unit (CTC Analytics AG, Zwingen, Switzerland) that samples directly from the headspace using a 1 mL airtight heated syringe, and an Agilent 490 gas chromatograph (GC, Agilent Technologies, Palo Alto, CA, USA). For raw milk analysis, according the method provided by Feng et al. (23) and Wang et al. (24). 5 mL of sample was placed in a 20 mL glass headspace-sampling vial. After incubation at 80°C for 20 min at 500 rpm, 500 μL headspace was injected into the GC-IMS (injection port temperature: 85°C). N_2_ (purity ≥ 99.999%) was used to drive the headspace into an FS-SE-54-CB capillary column (15 m × 0.53 mm) under isothermal conditions at 60 °C, and the GC was programmed as follows: 2 mL/min for 2 min, ramped to 10 mL/min within 8 min, then ramped to 100 mL/min within 10 min, and finally ramped to 150 mL/min within 5 min. The total GC run time was 25 min.

Volatile compound identification was performed according to the method provided by Li et al. (16). All analyses were performed in triplicate. N-ketones C4-C9 (Sinopharm Chemical Reagent Beijing Co., Ltd, P.R. China) were used as an external reference to calculate the volatile compound retention index (RI). The RI and drift time of the standard were compared with the GC × IMS library and NIST database to identify the volatile compounds.

### Statistical Analysis

The instrumental analysis software used to analyze the data was Laboratory Analytical Viewer (LAV) with three plug-ins (Reporter, Gallery Plot, and Dynamic PCA plug-in). LAV was used to view the analysis spectrum and perform qualitative analysis of volatile compounds, using the built-in NIST and IMS databases. The Reporter plug-in was used to make 3D-topographic, 2D-topographic, and difference spectrograms to analyze volatile compounds in different types of milk. The Gallery Plot plug-in used to draw Gallery Plot and Dynamic PCA plug-in were used for fingerprint and PCA. The results of VOCs data and *P*-value based on the data of VOCs was analyzed using one-way analysis of variance models in SAS (version 9.4, SAS Inst., Inc., Cary, NC, USA). The following statistical model was used:


$$\begin{array}{l} \text{Yij}=\text{μ}+\text{Ti}+\text{ εij}, \end{array}$$

where Yij represents the observed dependent variables, μ is the overall mean, Ti is the effect of treatment, and εij is the residual error. The significance level was declared at *P* < 0.05.

## Results and Discussion

### HS-GC–IMS Topographic Plots of Different Types of Raw Milk

**Figure 1:**
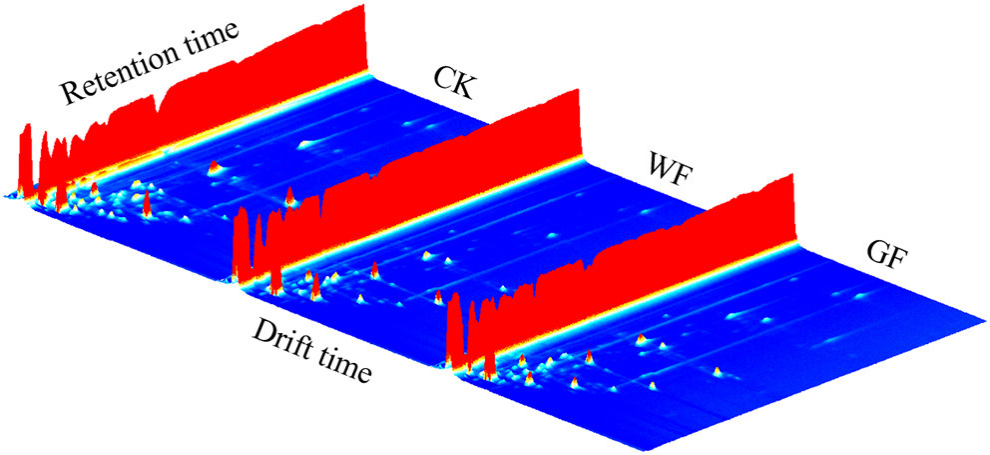
3D topographical visualization of raw milk from cows fed different diets. CK, basal diet (cows were not fed flaxseed); WF, whole flaxseed (dairy cows were fed WF: 1,500 g/d per cow); GF, ground flaxseed (dairy cows were fed GF: 1,500 g/d per cow).

In this study, HS-GC-IMS was used to investigate the VOCs distribution and further discriminate the raw milk from the cows with different diet. Due to the 2-dimensional nature of HS-GC-IMS measurements, the VOCs of sample were well-separated by two procedures at the ionized and drift regions. After the pre-separation by using GC's capillary column, the volatile substances were transported into the electric field of the IMS to separate again. The results are presented in the 3D topographical visualization. The volatile compounds detected in raw milk from cows fed different diets were similar, but intensity of the signal was slightly different (as showed in Figure 1). In the final, a total of 40 typical target compounds including three acids, six esters, 11 aldehydes, seven alcohols, 13 ketones were identified by using the GC × IMS Library in different raw milk samples (Table 1; Supplementary Figure 1). Previous studies have typically involved the use of HS-SPME-GCMS to analyze 32~33 volatile compounds in raw milk (13, 26). Hence, the number of VOCs reported in literatures was less than that of this study. This may due to the difference in analytical instruments between HS-GC-IMS and HS-SPME GCMS, HS-GC-IMS analysis has the potential ability to distinguish between monomers (M) and dimers (D) of target analytes, and dimers may play an important role in distinguishing raw milk from the cows feed different diets. Previous reported that benzaldehyde (D) and phenylacetaldehyde (D) were reliable markers of winter honey, and phenylethyl acetate dimer was reliable marker of *sapium* honey (27). In this study, 11 kinds of compound dimers [hexanoic acid (D), ethyl acetate (D), heptanal (D), 3-methylbutanal (D), 3-methylbutanol (D), ethanol (D), 2-heptanone (D), 2-pentanone (D), 2-butanone (D), 4-methyl-2-pentanone (D), acetoin (D)] were detected in three raw milks. And Heptanal (D), Ethanol (D), 2-Heptanone (D), 2-Pentanone (D), 2-Butanone (D), 4-Methyl-2-pentanone (D) showed different between the three milks (*P* < 0.05).

**Table 1:** Compounds identified in raw milk from cows fed different diets by GC-IMS (Mean ± SE).

						**Intensity (V)**			
**Volatiles**	**No**.	**Compounds**	**RI^**1**^**	**RT^**2**^**	**DT^**3**^**	**CK**	**WF**	**GF**	***P*-value**
Acids	6	Hexanoic acid (M)	998.1	581.6	1.310	1014.25 ± 345.44^a^	420.58 ± 112.08^b^	966.47 ± 459.61^a^	0.001
	7	Hexanoic acid (D)	999.9	584.4	1.644	123.62 ± 35.83	87.62 ± 6.13	123.86 ± 63.86	0.108
	35	2-Methylpropanoic acid	782.4	260.5	1.162	429.94 ± 115.23^a^	171.12 ± 37.30^c^	312.15 ± 119.44^b^	<0.001
Esters	4	Ethyl hexanoate	1012.8	606.0	1.341	102.01 ± 48.77^a^	58.45 ± 6.39^b^	80.54 ± 28.88^ab^	0.023
	11	3-Methylbutyl acetate	877.8	371.4	1.308	51.98 ± 14.59	42.11 ± 6.90	75.71 ± 63.23	0.142
	13	Ethyl butanoate	797.2	275.2	1.206	299.40 ± 205.34^a^	97.32 ± 15.81^b^	209.14 ± 140.99^ab^	0.015
	28	Ethyl Acetate (M)	607.2	147.6	1.095	576.03 ± 504.29	219.15 ± 37.75	563.94 ± 585.03	0.147
	29	Ethyl Acetate (D)	607.2	147.615	1.335	853.81 ± 80.4	150.33 ± 198.80	710.77 ± 1020.33	0.268
	34	Propyl acetate	712.7	201.	1.162	131.84 ± 74.81^b^	166.19 ± 40.33^b^	264.37 ± 95.98^a^	0.001
Aldehyde	8	Benzaldehyde	957.9	501.	1.148	159.00 ± 33.53	167.49 ± 14.13	180.13 ± 23.21	0.181
	1	Nonanal	1104.8	784.7	1.478	506.50 ± 83.37^b^	641.21 ± 74.88^a^	597.14 ± 112.62^a^	0.009
	3	Phenylacetaldehyde	1049.7	672.1	1.258	42.17 ± 4.37	41.52 ± 6.42	45.33 ± 13.42	0.602
	5	Octanal	1012.2	605.1	1.411	289.16 ± 77.82^b^	486.03 ± 127.99^a^	375.32 ± 108.47^b^	0.001
	14	Hexanal	793.6	271.5	1.255	472.89 ± 184.01^c^	1113.87 ± 271.48^a^	788.22 ± 425.70^b^	<0.001
	26	Heptanal (M)	898.0	400.4	1.337	232.63 ± 68.37^c^	438.0 ± 91.53^a^	342.77 ± 128.83^b^	<0.001
	27	Heptanal (D)	897.6	399.9	1.696	41.68 ± 9.74^b^	68.12 ± 20.2^a^	61.99 ± 27.66^b^	0.020
	32	3-Methylbutanal (M)	650.0	166.0	1.174	390.85 ± 157.8	225.26 ± 91.30	426.81 ± 292.92	0.072
	33	3-Methylbutanal (D)	645.7	164.1	1.407	114.65 ± 82.34	68.75 ± 30.76	178.90 ± 289.20	0.379
	38	Pentanal	694.7	188.8	1.185	146.92 ± 35.67^b^	254.89 ± 43.88^a^	213.31 ± 55.33^b^	<0.001
	40	Butanal	594.6	142.6	1.290	277.12 ± 253.25	84.31 ± 41.19	219.61 ± 269.93	0.140
Alcohols	12	1-Hexanol	868.8	359.2	1.326	216.10 ± 76.10	161.47 ± 52.46	248.87 ± 105.39	0.068
	24	3-Methylbutanol (M)	743.6	225.9	1.245	447.07 ± 247.99^a^	159.15 ± 38.68^b^	365.26 ± 364.86^ab^	0.049
	25	3-Methylbutanol (D)	735.4	219.2	1.500	121.34 ± 71.61	56.68 ± 11.05	118.92 ± 125.18	0.165
	30	Ethanol (M)	457.3	97.7	1.050	1568.48 ± 810.76^a^	117.06 ± 66.26^b^	1305.11 ± 868.20^a^	<0.001
	31	Ethanol (D)	465.7	100.0	1.132	309.06 ± 276.34^a^	11.05 ± 1.87^b^	247.00 ± 312.96^a^	0.025
	36	Linalool	1088.7	749.9	1.221	213.81 ± 31.69	209.69 ± 11.11	238.13 ± 41.33	0.101
	37	2-Hexanol	777.2	255.5	1.286	111.05 ± 46.87^a^	44.24 ± 8.29^b^	84.14 ± 36.18^a^	0.001
ketones	2	2-Nonanone	1097.9	769.6	1.409	386.42 ± 145.70	270.6 ± 105.50	388.9 ± 125.08	0.075
	9	2-Heptanone (M)	891.5	390.9	1.26047	2089.02 ± 301.82^a^	1549.46 ± 319.75^b^	1859.38 ± 321.12^a^	0.003
	10	2-Heptanone (D)	889.0	387.2	1.634	1520.65 ± 480.93^a^	765.00 ± 357.96^b^	1132.38 ± 416.17^ab^	0.002
	15	2-Pentanone (M)	685.8	183.3	1.120	1211.47 ± 135.09^a^	967.75 ± 135.43^b^	1064.04 ± 119.19^b^	0.001
	16	2-Pentanone (D)	683.4	182.0	1.370	1748.68 ± 581.72^a^	727.58 ± 249.59^b^	1088.62 ± 493.06^b^	<0.001
	17	2-Butanone (M)	584.9	138.8	1.062	1715.42 ± 373.90^a^	1103.58 ± 381.65^b^	1445.42 ± 365.84^ab^	0.004
	18	2-Butanone (D)	586.5	139.4	1.248	1393.12 ± 1030.04^ab^	1867.48 ± 1094.50^a^	732.19 ± 576.63^b^	0.034
	19	Acetone	503.0	110.8	1.119	6559.13 ± 1067.66	7144.04 ± 447.41	6962.74 ± 924.72	0.310
	20	4-Methyl-2-pentanone (M)	736.9	220.4	1.174	193.87 ± 79.73	274.68 ± 36.99	235.10 ± 92.98	0.067
	21	4-Methyl-2-pentanone (D)	733.8	218.0	1.483	144.39 ± 48.26^a^	79.04 ± 23.10^b^	155.68 ± 60.15^a^	0.002
	22	Acetoin (M)	714.6	203.1	1.060	1456.84 ± 990.83^a^	602.77 ± 139.75^b^	1125.42 ± 465.92^ab^	0.020
	23	Acetoin (D)	714.6	203.1	1.333	483.81 ± 676.34	180.41 ± 23.83	258.57 ± 1140.92	0.229
	37	2-Hexanone	783.8	261.8	1.188	114.17 ± 15.31	94.2 ± 9.07	108.21 ± 29.3	0.089

*(M), Monomer; (D), Dimer*.

a *Represents the retention index calculated using n-ketones C4–C9 as an external standard with a FS-SE-54-CB column*.

b *Represents the retention time in the capillary GC column*.

c *Represents the drift time in the drift tube*.

### Volatile Compounds in Different Types of Raw Milk

**Figure 2:**
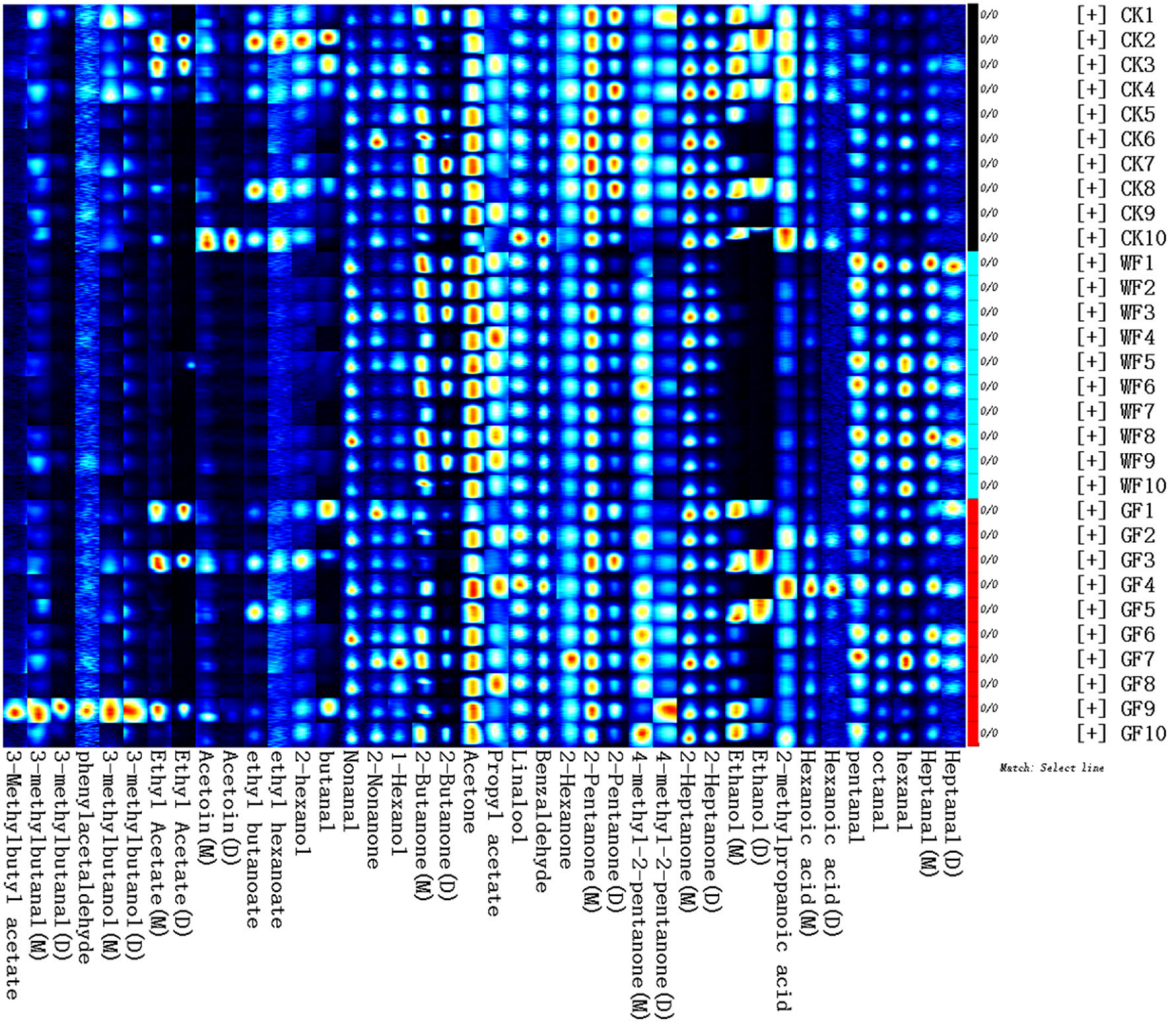
Gallery Plot in raw milk from cows fed different diets. (M), Monomer; (D), Dimer. CK, dairy cows with a basal diet (without flaxseed); WF, whole flaxseed (dairy cows were fed whole flaxseed: 1,500 g/d per cow); GF, ground flaxseed (dairy cows were fed ground flaxseed: 1,500 g/d per cow).

**Figure 3:**
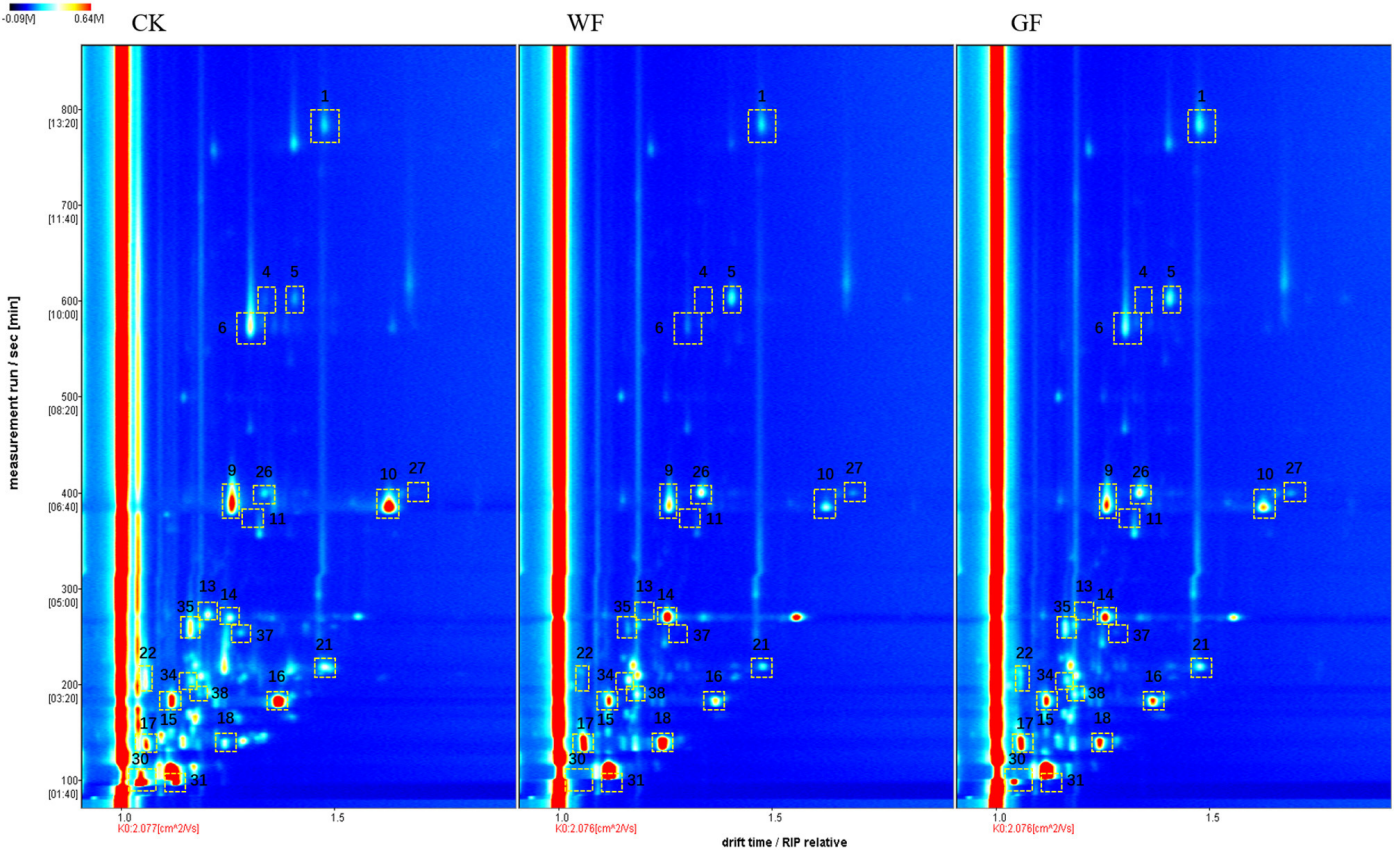
HS-GC–IMS spectra of raw milk from cows fed different diets. (M), Monomer; (D), Dimer. CK, basal diet (without flaxseed); WF, whole flaxseed (dairy cows were fed WF: 1,500 g/d per cow); GF, ground flaxseed (dairy cows were fed GF: 1,500 g/d per cow). 1: Nonanal, 4: Ethyl hexanoate, 5: Octanal, 6: Hexanoic acid (M), 9: 2-Heptanone (M), 10: 2-Heptanone (D), 11: 3-Methylbutyl acetate, 13: Ethyl butanoate, 14: Hexanal, 15: 2-Pentanone (M), 16: 2-Pentanone (D), 17: 2-Butanone (M), 18: 2-Butanone (D), 21: 4-Methyl-2-pentanone (D), 22: Acetoin (M), 26: Heptanal (M), 27: Heptanal (D), 30: Ethanol (M), 31: Ethanol (D), 34: Propyl acetate, 35: 2-Methylpropanoic acid, 37: 2-Hexanol, 38: Pentanal.

As shown in Figure 2, 40 volatile compounds were detected from the raw milk of the three group by GC-IMS NIST database. Each row represented a raw milk and each column meant a compound. It also can be seen from the Figure 2 that there are individual differences between cows in each group. The identified components in this experiment are shown in Table 1. In order to further analysis, the concentration of VOCs in different raw milk, the data was presented in 2D array full-size top view of HS-GC-IMS 3D-topographic plot (as showed in Figure 3). Each point on the spectrum represents a compound detected in the sample. Most of the signals were detected at retention times between 100 and 1,000 s and the drift time was between 1 and 1.8 ms. The color represents the intensity of the VOCs, and color from white to red mean the intensity turning higher.

As showed in Table 1; Figure 3, contents of two acid (hexanoic acid (M), 2-methylpropanoic acid), three esters (ethyl hexanoate, ethyl butanoate, propyl acetate), four alcohols (3-methylbutanol (M), ethanol (M), ethanol (D), 2-hexanol), seven ketones [2-heptanone (M), 2-heptanone (D), 2-pentanone (M), 2-pentanone (D), 2-butanone (M), 4-methyl-2-pentanone (D), acetoin (M)] were decreased and contents of six aldehydes [nonanal, octanal, hexanal, heptanal (M), heptanal (D), pentanal] were increased by WF supplementation compared to CK milk (*P* < 0.05). However, only five compounds including (2-methylpropanoic acid, 2-pentanone (M), 2-pentanone (D), 2-butanone (D) showed significantly different between GF and CK milk (*P* < 0.05). The concentration of propyl acetate in GF milk higher than CK and WF milk (*P* < 0.05).

Nonanal, heptanal, hexanal, octanal, and pentanal which usually detected in milk (15, 28, 29), which are lipid oxidation products of unsaturated fatty acids (UFA) degradation (30). Different kinds of aldehydes can provide different characteristics for milk. Nonanal usually has the flavor of fatty, citrus and green in milk (31); octanal usually has the flavor of citrus and floral (32); pentanal usually has the flavor of fermented, cardboard-like, bready, nutty (26); heptanal usually have the flavor of fatty, citrus and fishy (31, 33). Previous studies reported that feed flaxseed to laying hens could increase the flavor of fishy in egg which were less acceptable (34). Thus, the increase of heptanal may enhance unpleasant fishy smell in milk. In addition, previous reported that nonanal and hexanal were known as compounds which caused off-flavors in milk (26, 30). Octanal was also a compound derived off-flavors in milk (29). Moreover, the odor threshold values of aldehydes were low. In this experiment, compared with CK milk, the concentration of nonanal, heptanal, hexanal, octanal, and pentanal increase in WF milk, and nonanal increase in GF milk. Therefore, compared with GF supplementation, suppling with WF might cause more off-flavors in milk.

Hexanoic acid fatty has the flavor of fatty, sweaty, and like rancid butter (32). Santillo et al. (5) reported that supplementation with WF could reduce the concentration of C6:0 in milk. This study also found WF supplementation can decrease the concentration of Hexanoic acid. Ethanol is an important compound in milk, and which can be used to synthesize esters (34, 35), such as ethyl butanoate and ethyl hexanoate. Thus, lower concentration of ethanol may lead lower concentration of ethyl butanoate and ethyl hexanoate. Ethyl butanoate and ethyl hexanoate were common VOCs in milk, which can provide fruity flavor in raw milk. Previous reported esters may mask or attenuate the impact of off-flavor in milk (35). This study also found the lower concentration of ethanol, ethyl butanoate and ethyl hexanoate in WF milk, compared with CK milk. However, supplementation with GF showed no influence on the content of ethanol, ethyl butanoate and ethyl hexanoate in raw milk. Propyl acetate has the flavor of sweet, fruity, and celery (36), higher concentration of propyl acetate in GF group may provide more sweet, fruity. 2-Heptanone has flavor of fruity, spicy, sweet and 2-pentanone has flavor of sweet, fruity, they were the secondary oxidation products (29). Whole flaxseed supplementation can decrease the concentration of 2-Heptanone and 2-pentanone in raw milk.

In general, compared with GF supplementation, suppling with WF might cause more off-flavors in milk. WF supplementation in diet of dairy cows could increase fishy (heptanal) cardboard-like (pentanal) flavor in milk and decrease sweet (hexanoic acid, 2-heptanone), fruity (ethyl butanoate, ethyl hexanoate, 2-heptanone) flavor which may lead the milk less acceptable.

### Clustering Analysis of Different Raw Milk

**Figure 4:**
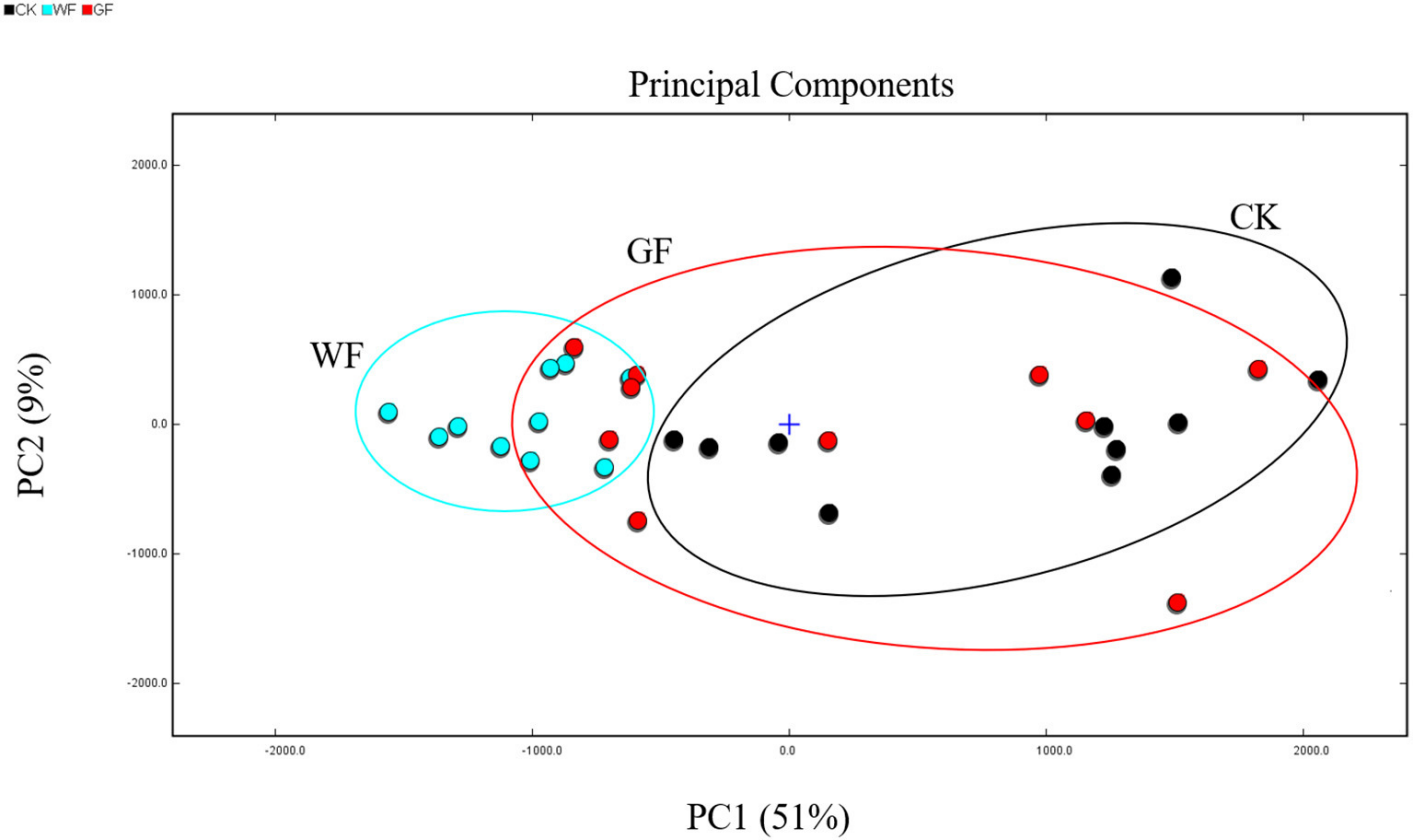
PCA scores plot of raw milk from cows fed different diets. Black points: CK milk; blue point: WF milk; red point: GF milk. CK, basal diet (without flaxseed); WF, whole flaxseed (dairy cows were fed WF: 1,500 g/d per cow); GF, ground flaxseed (dairy cows were fed GF: 1,500 g/d per cow).

PCA is a multivariate statistical analysis technique that is widely used to intuitively distinguish the samples of different treatments. PCA was used to compare the VOCs between CK, WF, and GF milk (as shown in Figure 4). And sum of the two principal components was 60% (PC1 = 51%; PC2 = 9%), and higher cumulative contribution rate, means the better the separation of the model. Wang et al. (21, 27) reported that the PCA model is considered a prefer separation model when the cumulative contribution rate reaches 60%. As showed in the PCA scores plot, the flavors in CK and WF milk were significantly different and could be well-distinguished using the value of PC1. On the PC1-axis, WF milk was distributed in the negative score values, whereas most CK milk was positive. However, it was difficult to distinguish between CK and WF milk using PC2 values. In addition, GF milk could not be distinguished from CK and WF milk. Thus, WF supplementation had a greater influence on VOCs than GF. These results revealed that changes in diet composition could influence the flavor of milk. Different types of milk can be distinguished through their characteristic volatile fingerprints established by HS-GC–IMS.

## Conclusions

This study demonstrates that the HS-GC-IMS is a useful tool to analyze the raw milk from cows fed different diets. In this study 40 target compounds were identified, including three acids, six esters, 11 aldehydes, seven alcohols, 13 ketones. Through analysis, flaxseed supplementation in diet of dairy cow could influence the VOCs in raw milk. Compared with CK milk, 22 compounds showed significantly different in WF milk, and five compounds in GF milk. Compared with GF supplementation, suppling with WF might cause more off-flavors and decrease the sweet (hexanoic acid) and fruity (2-heptanone) compound in milk. In addition, PCA based on the signal intensity of identified VOCs indicated that it was possible to distinguish between the CK and WF milk. In conclusion, compared with WF, GF supplementation in diet of dairy cow showed higher increase in n-3 PUFA in raw milk, and less influence in VOCs of raw milk. Afterwards, the effects of GF and WF supplementation in diet of dairy cows on the VOCs of raw milk could be further analyzed by sensory evaluation, thereby providing theoretical supports for the production of milk rich in n-3 PUFA. However, until now, the mechanism of different treatments of flaxseed on volatile substances in raw milk has not been clarified. Apart from the PUFA effect, the process of PUFA release may be central. Further study will focus on the use of metabolomic approaches to describe the process of PUFA release that influenced the VOC profile of raw milk.

## Data Availability Statement

The original contributions presented in the study are included in the article/Supplementary Material, further inquiries can be directed to the corresponding authors.

## Ethics Statement

The animal study was reviewed and approved by Animal Care and Use Committee of the Institute of Animal Science, Chinese Academy of Agricultural Sciences, Beijing, China.

## Author Contributions

GH: conceptualization, software, data curation, and writing original draft. NL: methodology. KL: data curation. JY: software. SZ: formal analysis. NZ: supervision. JZ: article modification. YZ: writing and reviewing. JW: project administration. All authors contributed to the article and approved the submitted version.

## Funding

The authors would like to thank the Agricultural Science and Technology Innovation Program (ASTIP-IAS12), the Modern Agro-Industry Technology Research System, P.R. China, and the Scientific Research Project for Major Achievements from the Agricultural Science and Technology Innovation Program (CAAS-ZDXT2019004).

## Conflict of Interest

The authors declare that the research was conducted in the absence of any commercial or financial relationships that could be construed as a potential conflict of interest.

## Publisher's Note

All claims expressed in this article are solely those of the authors and do not necessarily represent those of their affiliated organizations, or those of the publisher, the editors and the reviewers. Any product that may be evaluated in this article, or claim that may be made by its manufacturer, is not guaranteed or endorsed by the publisher.
