# Supplementary material for: Effect of Flaxseed Supplementation in Diet of Dairy Cow on the Volatile Organic Compounds of Raw Milk by HS-GC–IMS
Source: Front Nutr. 2022 Feb 14;9:831178. doi: 10.3389/fnut.2022.831178 (PMC8884162; doi:10.3389/fnut.2022.831178)
Supplement: Supplementary file 1 [file Image_1.pdf]

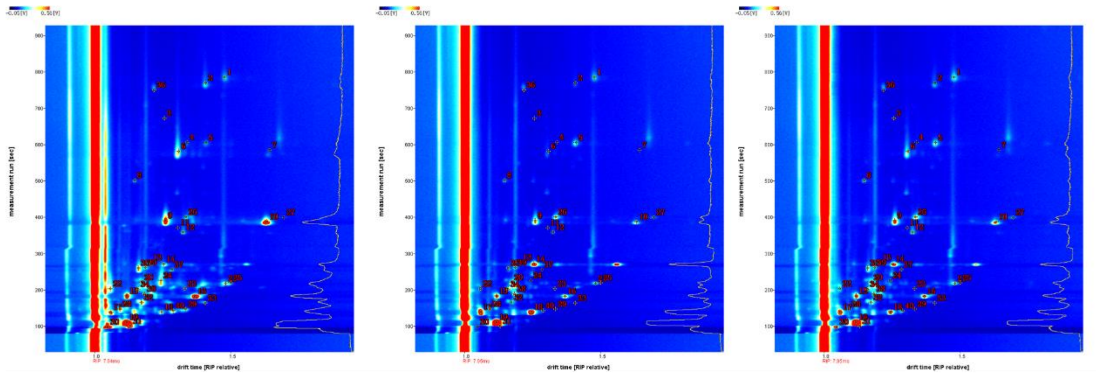

CK Group WF Group GF Group

Figure S1. Topographic plots of HS-GC-IMS spectra with the selected markers obtained with raw milk from different group.
